# Whole genome analysis of selected human and animal rotaviruses identified in Uganda from 2012 to 2014 reveals complex genome reassortment events between human, bovine, caprine and porcine strains

**DOI:** 10.1371/journal.pone.0178855

**Published:** 2017-06-22

**Authors:** Josephine Bwogi, Khuzwayo C. Jere, Charles Karamagi, Denis K. Byarugaba, Prossy Namuwulya, Frederick N. Baliraine, Ulrich Desselberger, Miren Iturriza-Gomara

**Affiliations:** 1 EPI laboratory, Uganda Virus Research Institute, Entebbe, Uganda; 2 Department of Paediatrics and Child Health, College of Health Sciences, Makerere University, Kampala, Uganda; 3 Department of Clinical Infection, Microbiology and Immunology, Institute of Infection and Global Health, University of Liverpool, Liverpool, United Kingdom; 4 Malawi-Liverpool-Wellcome Trust Clinical Research Programme / Department of Medical Laboratory Sciences, University of Malawi, College of Medicine, Blantyre, Malawi; 5 Department of Microbiology, College of Veterinary Medicine and Biosecurity, Makerere University, Kampala, Uganda; 6 Department of Biology and Kinesiology, LeTourneau University, Longview, Texas, United States of America; 7 Department of Medicine, University of Cambridge, Cambridge, United Kingdom; Universidad Nacional de la Plata, ARGENTINA

## Abstract

Rotaviruses of species A (RVA) are a common cause of diarrhoea in children and the young of various other mammals and birds worldwide. To investigate possible interspecies transmission of RVAs, whole genomes of 18 human and 6 domestic animal RVA strains identified in Uganda between 2012 and 2014 were sequenced using the Illumina HiSeq platform. The backbone of the human RVA strains had either a Wa- or a DS-1-like genetic constellation. One human strain was a Wa-like mono-reassortant containing a DS-1-like VP2 gene of possible animal origin. All eleven genes of one bovine RVA strain were closely related to those of human RVAs. One caprine strain had a mixed genotype backbone, suggesting that it emerged from multiple reassortment events involving different host species. The porcine RVA strains had mixed genotype backbones with possible multiple reassortant events with strains of human and bovine origin.Overall, whole genome characterisation of rotaviruses found in domestic animals in Uganda strongly suggested the presence of human-to animal RVA transmission, with concomitant circulation of multi-reassortant strains potentially derived from complex interspecies transmission events. However, whole genome data from the human RVA strains causing moderate and severe diarrhoea in under-fives in Uganda indicated that they were primarily transmitted from person-to-person.

## Introduction

Rotaviruses belong to the genus *Rotavirus* of the family *Reoviridae*, comprising nine species (groups) designated as A, B, C, D, E, F, G, H and I [1, 2] and possibly a tenth species J [3]. Group A rotaviruses (RVA) are a leading cause of diarrhoea in children and young animals worldwide [4, 5]. In children, the infection may lead to severe dehydration and may cause death if the condition is not well managed [6]. Rotavirus infections in animals may affect productivity and have important economic consequences [7].

The rotavirus genome consists of eleven segments of double-stranded RNA (dsRNA). All RNA segments, with the exception of segment 11, are monocistronic, encoding either structural viral proteins (VP1 to VP4, VP6 and VP7) or non-structural proteins (NSP1 to NSP4). Genome segment 11 codes for two proteins: NSP5 and NSP6 [8]. Rotaviruses can be differentiated by a dual classification system, based on the two outer capsid proteins, VP7 and VP4, that determine the G (VP7, glycoprotein) and P (VP4, protease sensitive) genotypes, respectively [9]. At least 35 G types and 50 P types have so far been identified in humans and animals (rega.kuleuven.be/cev…/virus classification/newgenotype) [10, 11].

Globally, genotypes G1, G2, G3, G4, G9 and G12 in combination with P[4], P[6] or P[8] constitute more than 90% of the circulating human RVA strains [12]. The most common combinations of the G and P genotypes are G1P[8], G2P[4], G3P[8], G4P[8], G9P[8] and G12P[8] [12, 13]. However, regional variability has been observed. In Africa, RVA genotypes such as G8P[6] and G8P[8] are highly prevalent but uncommon elsewhere [14]. These uncommon rotavirus strains are thought to have arisen from host interspecies transmission [15, 16].

More recently, RVAs have been classified based on the sequence diversity of all 11 segments, assigning specific genotypes according to established nucleotide homology cut-off values [10, 17, 18]. This classification system, combined with whole genome sequencing and phylogenetic analysis, has been used to trace interspecies transmission events and potential origins of new and emerging strains [10, 19, 20].

Interspecies transmission of RVAs is thought to be an important contributor to rotavirus evolution, contributing to the diversity of viruses in both humans and animals [5, 8]. Other mechanisms for rotavirus evolution include accumulation of point mutations, gene reasortment or rearrangement, and gene recombination [5]. Genome evolution may occur either intragenically or intergenogroup [21, 22]. The combination of interspecies transmission and reassortment between RVAs of different species can lead to the emergence and spread of novel rotavirus strains [23].

Globally, few RVA co-surveillance studies in animals and humans in the same geographical region have been carried out. A study in the Netherlands found no evidence of interspecies transmission, the animal and human rotaviruses appeared to evolve separately [24]. By contrast, a study in Slovenia found interspecies transmission, with evidence of transmission from pigs to humans [25]. A study in Southern India found evidence of human-to-animal transmission of a G2 RVA strain [26]. Another study in Northern India found possible reassortment between genes of animals and human RVAs resulting in circulation of unusual rotavirus genotypes [27].

None of the above studies characterised whole genomes of RVAs from different species, hence provided only a partial picture of a potentially more widespread phenomenon. In the present study, whole genomes of RVAs identified from faecal samples of humans and animals living in the same region in Uganda during 2012–2014 were sequenced and analysed in order to investigate possible interspecies transmission events of RVAs in this setting.

## Materials and methods

### Ethical approval

This study was approved by the Research and Ethics Committees of the School of Medicine, College of Health Sciences, Makerere University (REF 2011–061); Uganda Virus Research Institute (GC/127/319); Mulago National Referral Hospital; St. Francis Hospital Nsambya; and Uganda National Council for Science and Technology (HS 1186). The caretakers/guardians of the children gave written consent for the children to participate in the study. In addition, the animal owners gave written consent for their animals to be included in the study.

### Human and animal recruitment, and sample collection

**Fig 1:**
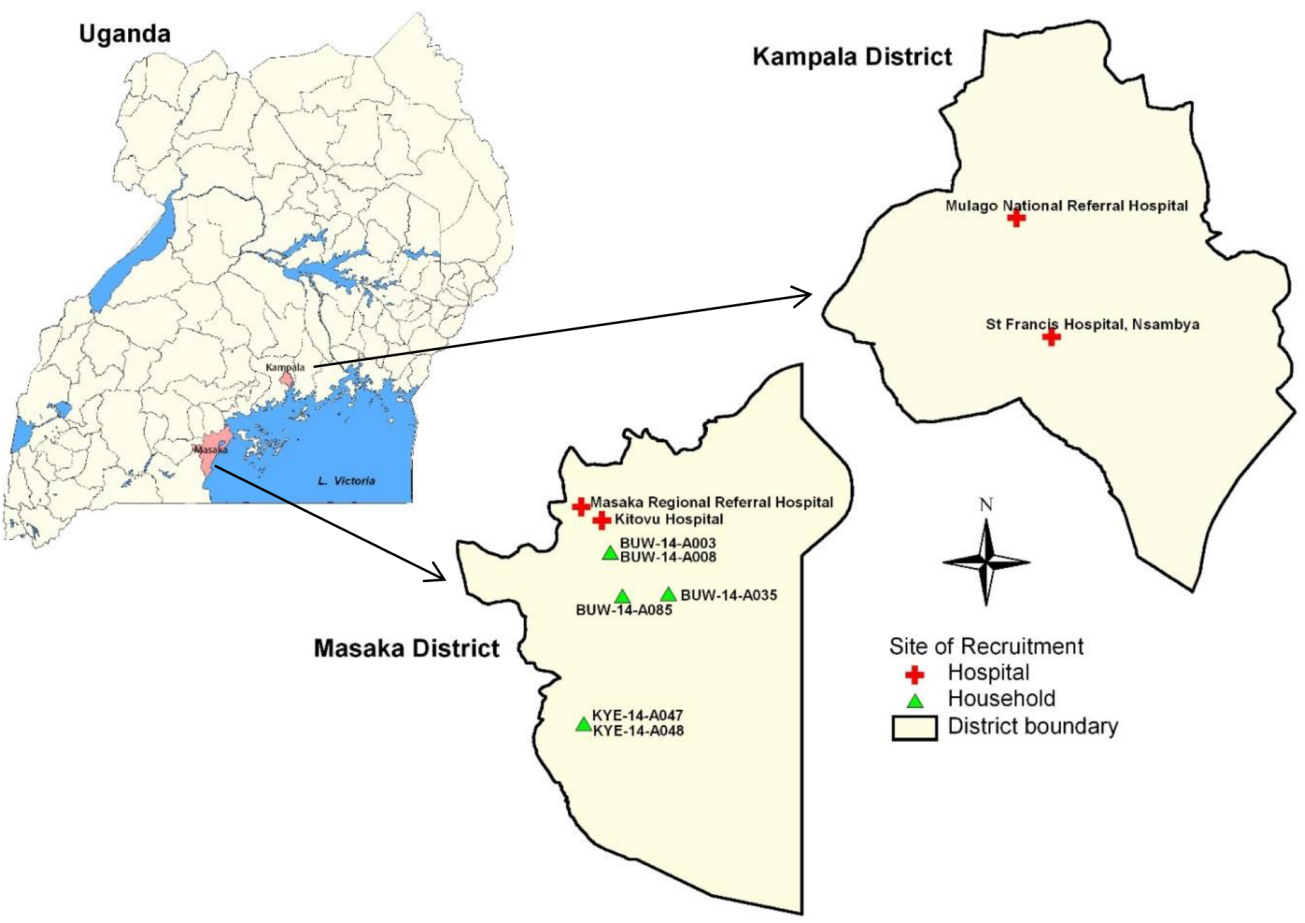
The maps of Uganda, Kampala and Masaka districts showing the hospitals and households at which the study children and animals (rotavirus positives that were sequenced) were recruited respectively.

Human stool samples were collected from children under-five years old hospitalised with acute diarrhoea in four hospitals located in Kampala and Masaka districts in central Uganda (Fig 1). The study was carried out from September 2012 through September 2013. The stools were investigated as previously described [28]. Eighteen out of 208 human RVA-positive samples were selected for whole genome sequencing. Selection was based on the availability of sufficient material and adequate viral load for unbiased sequencing directly from the stool sample, presence of G and P types found in animal RVAs, or the presence of unusual G and/or P types.

Animal stool samples were collected from 116 symptomatic (with history of diarrhoea in previous two weeks) and 984 asymptomatic (without history of diarrhoea in previous two weeks) domestic animals (cattle, goats and pigs) in homes located in Bukoto county, Masaka district from December 2013 through January 2014 (Fig 1, S1 Table). Out of the 41 RVA-positive animal samples whole genome analysis was possible on six samples (one bovine, one caprine and four porcine). Among these, only the bovine RVA was associated with a history of diarrhoea lasting four days in the two weeks prior to sample collection.

### Rotavirus dsRNA extraction, cDNA synthesis and amplification from human and animal samples

Rotavirus dsRNA was extracted from human stool suspension (approximately 100 mg of stool were suspended in 200 μl of PBS or 200 μl of semi-formed stool was mixed with 150 μl of PBS) using TRIZOL LS Reagent (Invitrogen, Carlsbad, CA, USA). The procedure of extraction was as previously described [29]. The guanidinium isothiocyanate silica method was used to extract rotavirus dsRNA from 10% faecal suspensions in PBS for all animal samples [30].

Oligonucleotide ligation to enable cDNA synthesis, and unbiased PCR amplification and purification of the entire rotavirus genome were carried out on the human samples that yielded more than 2 ng/μl dsRNA as described previously [29, 31]. The purified rotavirus cDNA PCR amplicons were subjected to standard bar-coding and library construction for Illumina sequencing using the Nextera XT DNA Library Preparation Kit following the manufacturer’s recommendations (Illumina Inc., CA, USA). For all animal samples and human samples that yielded less than 2 ng/μl dsRNA, the ScriptSeq v2 RNA-Seq Library Preparation Kit (Epicentre, Chicago, IL, USA) was used, following the manufacturer’s instructions with the slight modification of an initial denaturation step (95°C for 5 min). Each library was indexed with Illumina compatible barcodes to allow multiplexing (http://dx.doi.org/10.17504/protocols.io.h4vb8w6). The quality of the libraries was assessed using the VP6-specific qPCR [32] and the 2100 Bioanalyzer (Agilent Technologies, Santa Clara, CA, USA). The libraries were quantified with the Qubit dsDNA High Sensitive assay (Life Technologies, Carlsbad, CA, USA), and sequenced using the HiSeq 2500 Illumina platform at the Centre for Genomic Research, University of Liverpool, UK.

### Nucleotide sequence assembly, genotype assignment and phylogenetic analyses

Illumina adapter sequences were trimmed from the raw Fastq sequence data using Cutadapt version 1.2.1 and Sickle version 1.2 software [33]. Both *de novo* and mapping assembly tools embedded in Geneious software [34] were employed to generate consensus sequences for all analysed strains. To ensure that the multiple sequences detected in some of the samples were not due to assembly artifacts, sequence reads that had more than one contig were mapped separately to both Wa and DS-1 rotavirus prototype strains using both medium and high custom sensitivity parameters where only sequence reads with more than 80% overlap identity were used to build the consensus. Mixed populations were only accepted as true populations when the two consensus sequences generated through mapping and *de novo* assemblers were identical, could be translated to a functional protein without need for editing and had coverage of at least 200. The presence of multiple sequences in a single specimen was confirmed at the J. Craig Venter Institute by the Virology Project Team who blindly and independently assembled the sequence reads on CLC command-line assembly module (CLC Bio’s clc_novo assemble and CLC Bio’s clc_ref_assemble_long_program) [35].

RotaC version 2 (http://rotac.regatools.be/) [36], a classification tool for RVAs, was used to assign genotypes to all eleven genome segments. The nucleotide sequences generated in this study were deposited into the NCBI GenBank under the accession numbers KX632243-632352, KX655437-KX655538, KX988264-KX988283, KY055416-KY055437, KY077640-KY077650 (S2 Table).

Phylogenetic analysis was conducted using MEGA version 6.06 [37]. Multiple alignments of sequences from the study strains and reference strains from GenBank were carried out using the Multiple Sequence Comparison by Log-Expectation (MUSCLE) software [38]. The phylogenetic trees were constructed using the Maximum-Likelihood method with the best-fit substitution models. The substitution models that best fitted the sequence data were determined using the corrected Akaike Information Criterion (AICc). The models used in this study were: GTR+G+I for VP1, VP2 and VP3; T92+G for VP4, VP6, VP7, NSP1, NSP3 and NSP5; TN93+G+I for NSP2; and HKY +G for NSP4. The bootstrap (1000 replicates) values were used to determine the reliability of each node in the tree. The lineages for VP4 P[6] lineage I, P[4] lineage II and IV, P[8] lineage III, were assigned as previously suggested [16, 19, 39–42]. No literature was found with regard to the classification of P[1], P[7] and P[13] genes into lineages. The lineages for VP7: G1, G3, G6, G8, G9, G12 were assigned as previously suggested [39, 42–45]. Nucleotide distance matrices for each of the characterized RVA genomes were determined using BioEdit program [46].

## Results

The Illumina Hiseq sequencing yielded mean read lengths of 72.3 (SD 35) -119.1(SD 16.0) bp for the human and 34.0 (SD 20.5) -79.4 (SD 32.4) bp for animal RVA strains. The maximum expected read length was 125 bp. Complete nucleotide sequences were obtained for all the 11 segments of the 18 human strains and one bovine strain. Partial sequences were obtained for some genome segments of the porcine and caprine rotavirus strains (S3 Table). The fragments of the partial sequences ranged from 33.1% to 99.8% of the expected gene lengths (S3 Table). Nonetheless, these sequence lengths were adequate for assigning genotypes (Table 1).

**Table 1:** Whole genome constellation of characterised human and animal rotavirus strains circulating in Uganda, 2012–2014.

Species	Strain Nomenclature	VP7	VP4	VP6	VP1	VP2	VP3	NSP1	NSP2	NSP3	NSP4	NSP5
Human	RVA/Human-wt/UGA/KTV-13-023/2012/G12P[6]	G12	P[6]	I1	R1	C1	M1	A1	N1	T1	E1	H1
Human	RVA/Human-wt/UGA/MUL-13-183/2013/G12P[6]	G12	P[6]	I1	R1	C1	M1	A1	N1	T1	E1	H1
Human	RVA/Human-wt/UGA/NSA-13-043/2013/G9P[8]	G9	P[8]	I1	R1	C1	M1	A1	N1	T1	E1	H1
Human	RVA/Human-wt/UGA/MUL-13-163/2013/G9P[8]	G9	P[8]	I1	R1	C1	M1	A1	N1	T1	E1	H1
Human	RVA/Human-wt/UGA/MUL-12-147/2012/G9P[8]	G9	P[8]	I1	R1	C1	M1	A1	N1	T1	E1	H1
Human	RVA/Human-wt/UGA/MUL-12-093/2012/G9P[8]	G9	P[8]	I1	R1	C1	M1	A1	N1	T1	E1	H1
Human	RVA/Human-wt/UGA/MUL-13-285/2013/G9P[8]	G9	P[8]	I1	R1	C1	M1	A1	N1	T1	E1	H1
Human	RVA/Human-wt/UGA/MUL-13-157/2013/G1P[8]	G1	P[8]	I1	R1	C2	M1	A1	N1	T1	E1	H1
Human	RVA/Human-wt/UGA/MUL-12-104/2012/G3P[6]	G3	P[6]	12	R2	C2	M2	A2	N2	T2	E2	H2
Human	RVA/Human-wt/UGA/MUL-13-308/2013/G8P[6]	G8	P[6]	I2	R2	C2	M2	A2	N2	T2	E2	H2
Human	RVA/Human-wt/UGA/MUL-13-166/2013/G3P[6]	G3	P[6]	I2	R2	C2	M2	A2	N2	T2	E2	H2
Human	RVA/Human-wt/UGA/MUL-13-171/2013/G3P[6]**	G3/G3	P[6]/P[6]	I2	R2/R2	C2	M2	A2	N2	T2	E2	H2
Human	RVA/Human-wt/UGA/MUL-13-204/2013/G8P[6]	G8	P[6]	I2	R2	C2	M2	A2	N2	T2	E2	H2
Human	RVA/Human-wt/UGA/MUL-13-496/2013/G8P[4]	G8	P[4]	I2	R2	C2	M2	A2	N2	T2	E2	H2
Human	RVA/Human-wt/UGA/MUL-12-117/2012/G3P[6]	G3	P[6]	I2	R2	C2	M2	A2	N2	T2	E2	H2
Human	RVA/Human-wt/UGA/MUL-13-160/2013/G8P[4]	G8	P[4]	I2	R2	C2	M2	A2	N2	T2	E2	H2
Human	RVA/Human-wt/UGA/MSK-13-048/2013/G9P[8]	G9	P[8]	I2	R2	C2	M2	A2	N2	T2	E2	H2
Human	RVA/Human-wt/UGA/MUL-13-427/2013/G8P[4]	G8	P[4]	12	R2	C2	M2	A2	N2	T2	E2	H2
Goat	RVA/Goat-wt/UGA/BUW-14-A085/2014/G6P[1]	G6	P[1]	I2	R2	C2	M2	A11	N2	T6	E2	H3
Cattle	RVA/Cow-wt/UGA/BUW-14-A035/2014/G12P[8]	G12	P[8]	I1	R1	C1	M1	A1	N1	T1	E1	H1
Pig	RVA/Pig-wt/UGA/BUW-14-A008/2014/G12P[8]	G12	P[8]	I1	R1	C1	M1	A8	N1	T1	E1	H1
Pig	RVA/Pig-wt/UGA/BUW-14-A003/2014/G3P[13]	G3	P[13]	I1	R1	C1	M1	A8	N1	T7	E1	H1
Pig	RVA/Pig-wt/UGA/KYE-14-A047/2014/G3P[13]	G3	P[13]	I1	R1	C1	M1	A8	N1	T1	E1	H1
Pig	RVA/Pig-wt/UGA/KYE-14-A048/2014/G3P[13]	G3	P[13]	I1	R1	C1	M1	A8	N1	T1	E1	H1

** Sample contained mixed sequences of the same genotype in VP1, VP4 and VP7 genes

Colours represent the genome constellation: Green(Wa-like), Red(DS-1 like), White(non 1, non 2 genotype)

VP: viral structural protein, NSP: Viral non structural protein

### Whole genome classification of the analysed rotavirus strains

#### Complete genotype constellation of human strains

All but the genome segments encoding VP7 and VP4 for seven of human rotavirus strains (KTV-13-023, MUL-13-183, NSA-13-043, MUL-13-163, MUL-12-147, MUL-12-093 and MUL-13-285) had a Wa-like genotype constellation (-I1-R1-C1-M1-A1-N1-T1-E1-H1). One of the human Wa-like RVAs strain, MUL-13-157, contained a DS-1-like VP2 gene and was therefore classified as Wa-DS-1-like mono-reassortant (Table 1). The non-G and non-P genes of the other 10 human rotaviruses (MUL-12-104, MUL-13-308, MUL-13-166, MUL-13-171, MUL-13-204, MUL-13-496, MUL-12-117, MUL-13-160, MSK-13-048 and MUL-13-427) were assigned a DS-1-like genotype constellation (-I2-R2-C2-M2-A2-N2-T2-E2-H2), and hence were classified as DS-1-like human strains (Table 1).

Two distinct complete gene sequences of the same genotype were generated for genome segments encoding VP7, VP4 and VP1 for human rotavirus strain MUL-13-171, compatible with a mixed infection with two variant strains, by contrast, single sequences were generated for the remaining eight genome segments (Table 1).

#### Complete genotype constellation of animal strains

The characterised bovine strain had a Wa-like gene constellation. The G3P[13] porcine strains had predominantly Wa-like gene constellation, with the exception of the NSP1 gene (A8), and also the NSP3 gene in one of the strains (T7). The G12P[8] porcine strain also had a predominant Wa-like gene constellation with the exception of the NSP1 gene (A8). The G6P[1] caprine strain had a predominantly DS-1-like gene constellation with the exception of the genes encoding NSP1, NSP3 and NSP5 (Table 1).

### Phylogenetic analysis

In order to identify the relationships among the RVA strains detected from human and animal species in Uganda and investigate potential origin and evidence of interspecies transmission, phylogenetic analyses were conducted for each gene of the investigated RVA strains and compared with cogent RVA sequences available in the GenBank database.

In all the genes, except VP4 and VP7, the human strains clustered with Wa-like and DS-1 like human strains found in Africa including Democratic Republic of Congo (DRC), Tanzania, and Kenya which are neighbouring countries to Uganda (Fig 2, Fig 3, Figs 4–6, S1–S4 Figs) [16, 19, 29, 31, 39, 47–51].

**Fig 2:**
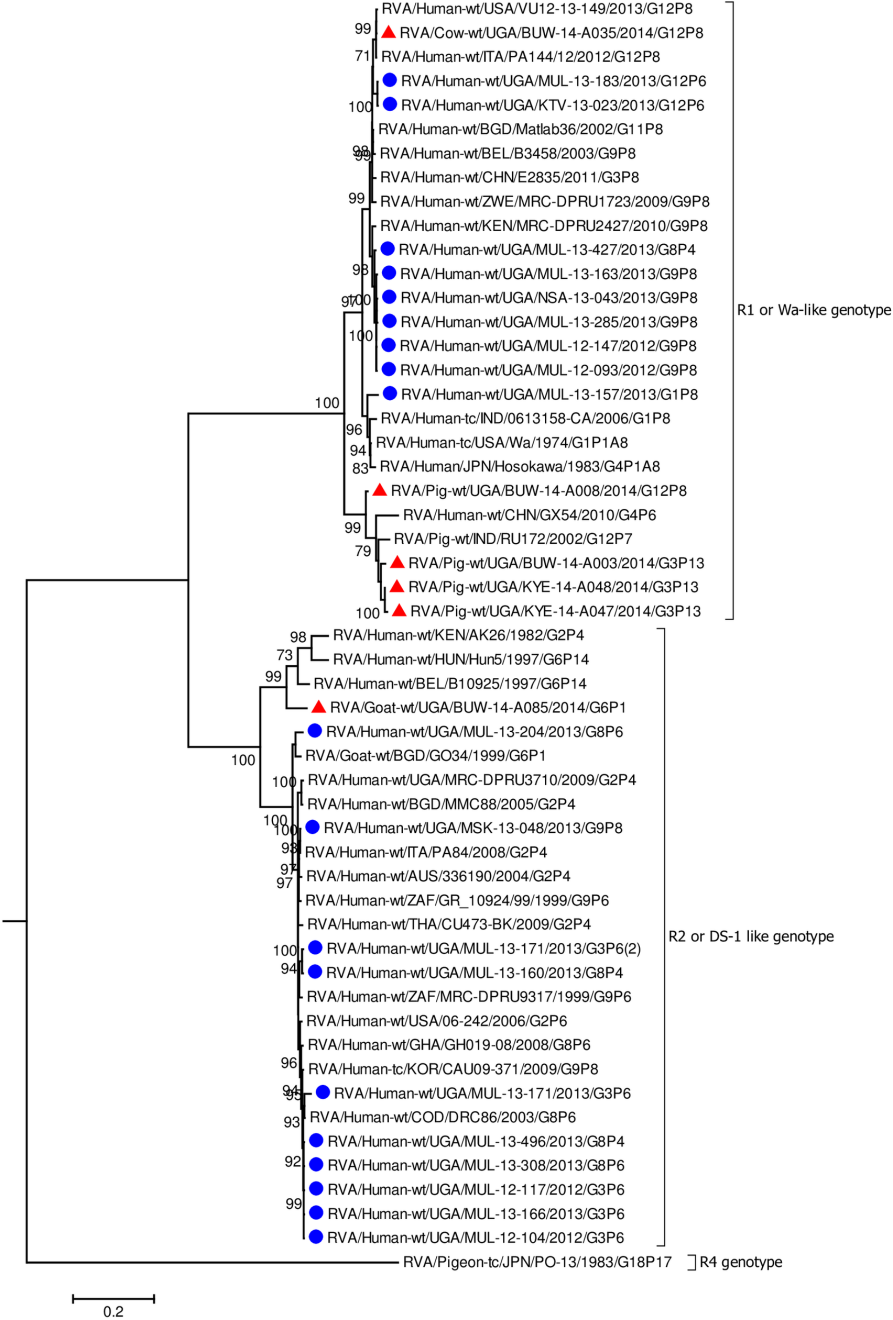
VP1 gene (segment 1). Maximum Likelihood phylogenetic trees of nucleotide sequences of rotavirus genome segment 1 of humans and animal RVA strains circulating in Uganda, 2012–2014. Bootstrap values above 70 are shown for 1000 replicates. The Ugandan human strains are labelled with blue circles and the Ugandan animal strains with red triangles. The Pigeon strain RVA/Pigeon-tc/PN/PO-13/1983/G18P[17] served as the outgroup. The scale bar at the bottom of the tree calibrates the genetic distance expressed as nucleotide substitution per site.

**Fig 3:**
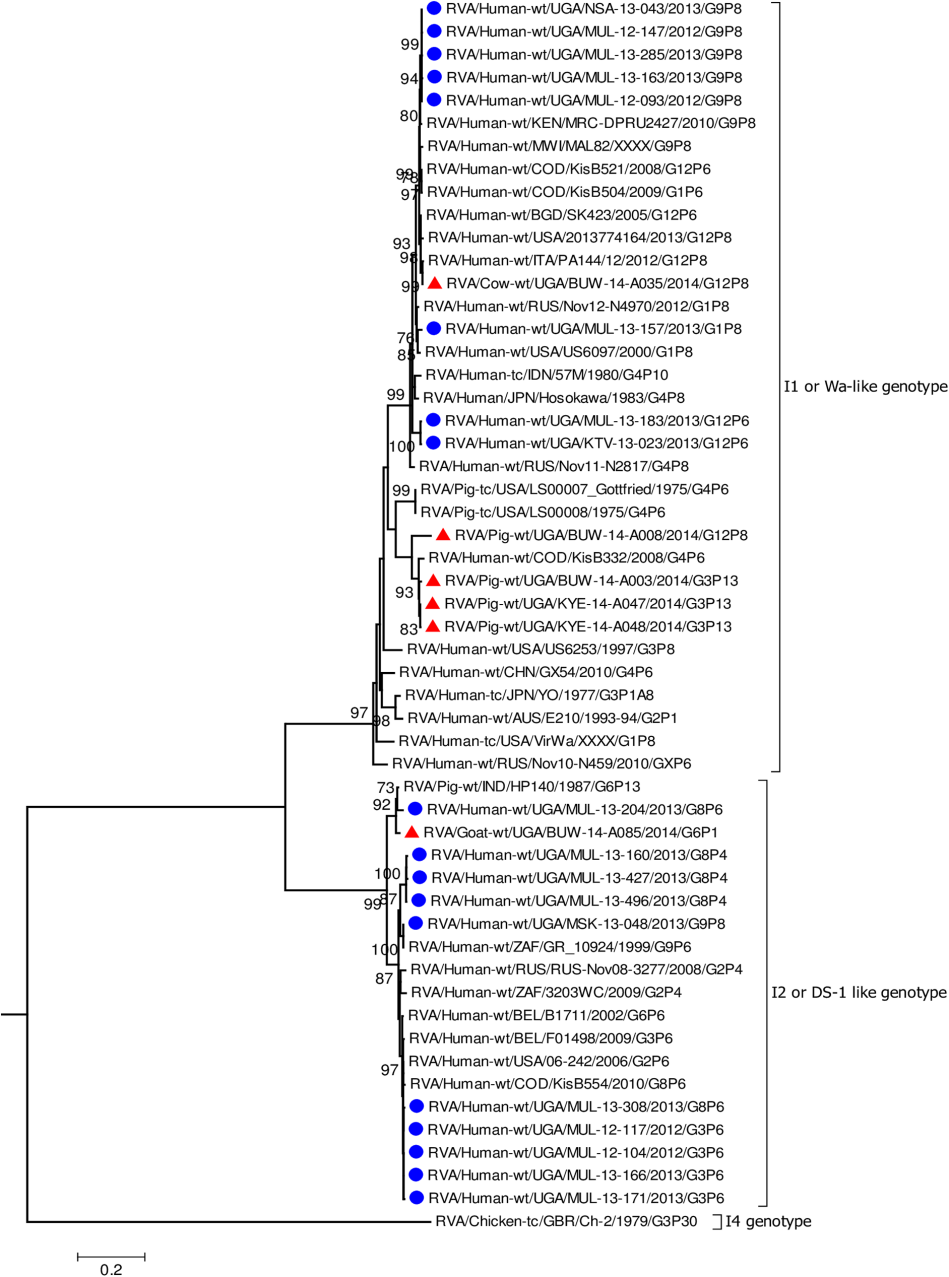
VP6 gene (segment 6). Maximum Likelihood phylogenetic trees of nucleotide sequences of rotavirus genome segment 6 of human and animal RVA strains circulating in Uganda, 2012–2014. Bootstrap values above 70 are shown for 1000 replicates. The Ugandan human strains are labelled with blue circles and the Ugandan animal strains with red triangles. Chicken strain RVA/Chicken-tc/GBR/Ch-2/1979/G3P[30] served as the outgroup. The scale bar at the bottom of the tree calibrates the genetic distance expressed as nucleotide substitution per site.

**Fig 4:**
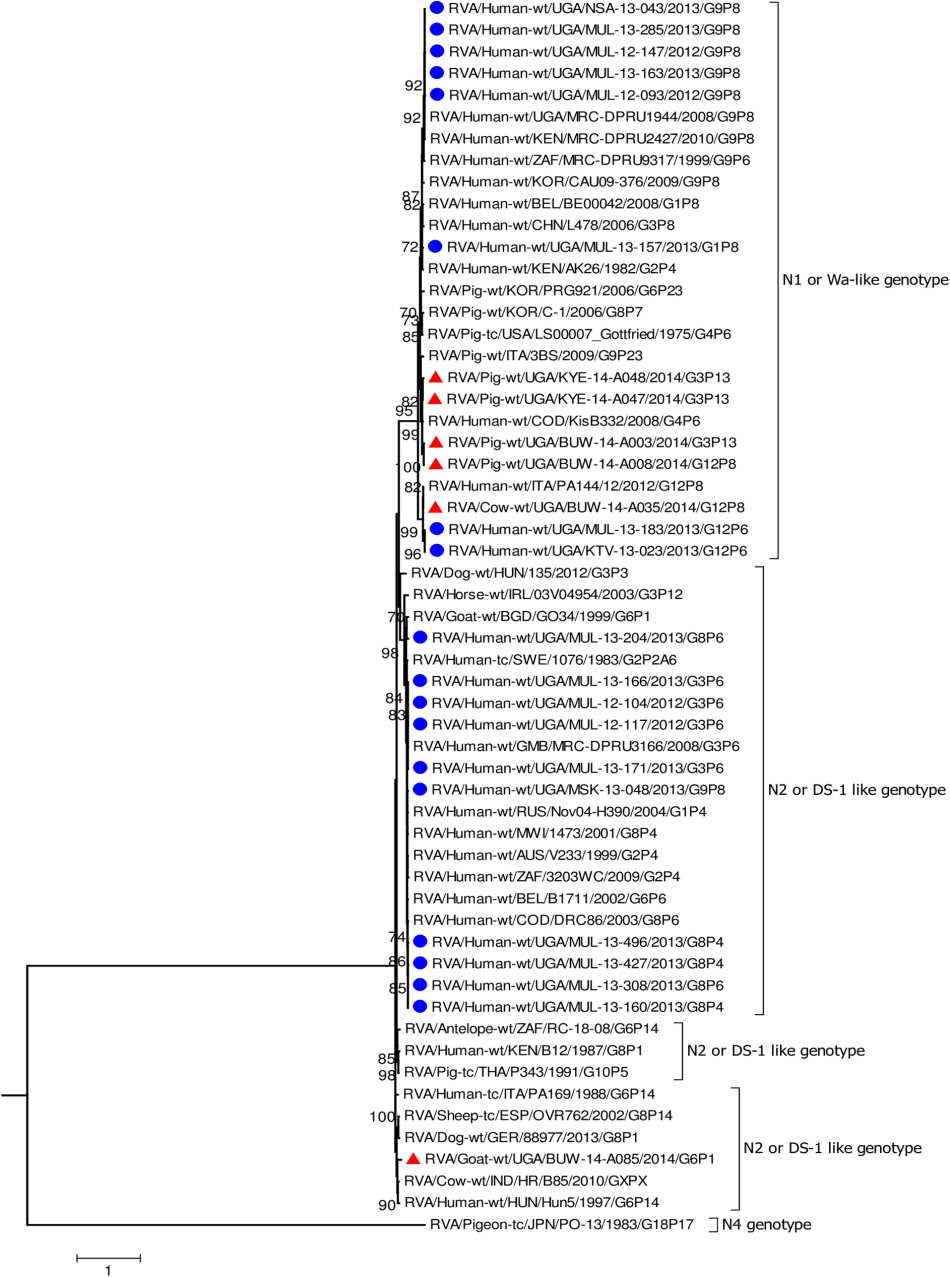
NSP2 gene (segment 8). Maximum Likelihood phylogenetic trees of nucleotide sequences of genome segment 8 of human and animal RVA strains circulating in Uganda, 2012–2014. Bootstrap values above 70 are shown for 1000 replicates. The Ugandan human strains are labelled with blue circles and the Ugandan animal strains with red triangles. Pigeon strain RVA/Pigeon-tc/PN/PO-13/1983/G18P[17] served as the outgroup. The scale bar at the bottom of the tree calibrates the genetic distance expressed as nucleotide substitution per site.

**Fig 5:**
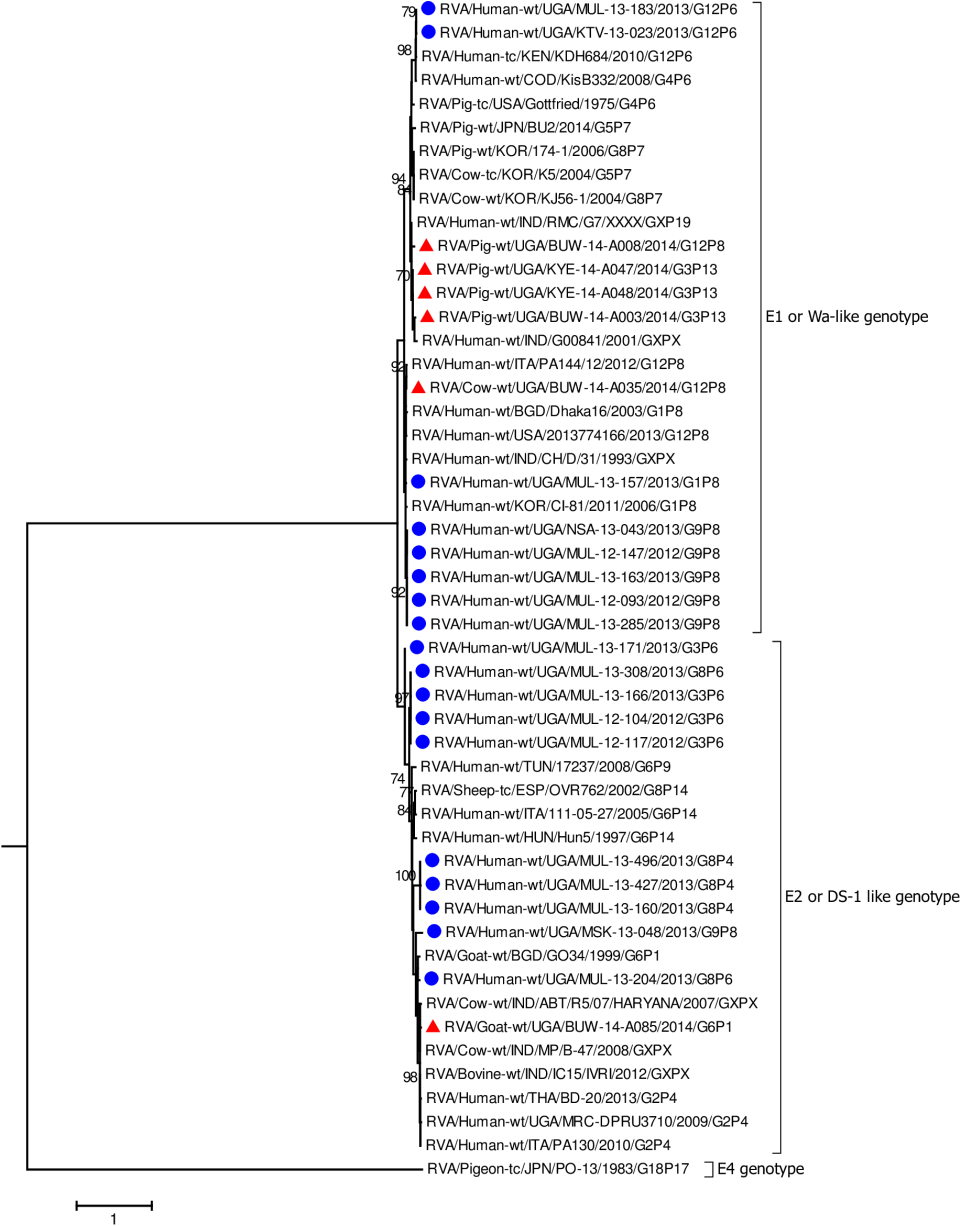
NSP4 gene (segment 10). Maximum Likelihood phylogenetic trees of nucleotide sequences of genome segment 10 gene of human and animal RVA strains circulating in Uganda, 2012–2014. Bootstrap values above 70 are shown for 1000 replicates. The Ugandan human strains are labelled with blue circles and the Ugandan animal strains with red triangles. Pigeon strain RVA/Pigeon-tc/PN/PO-13/1983/G18P[17] served as the outgroup. The scale bar at the bottom of the tree calibrates the genetic distance expressed as nucleotide substitution per site.

**Fig 6:**
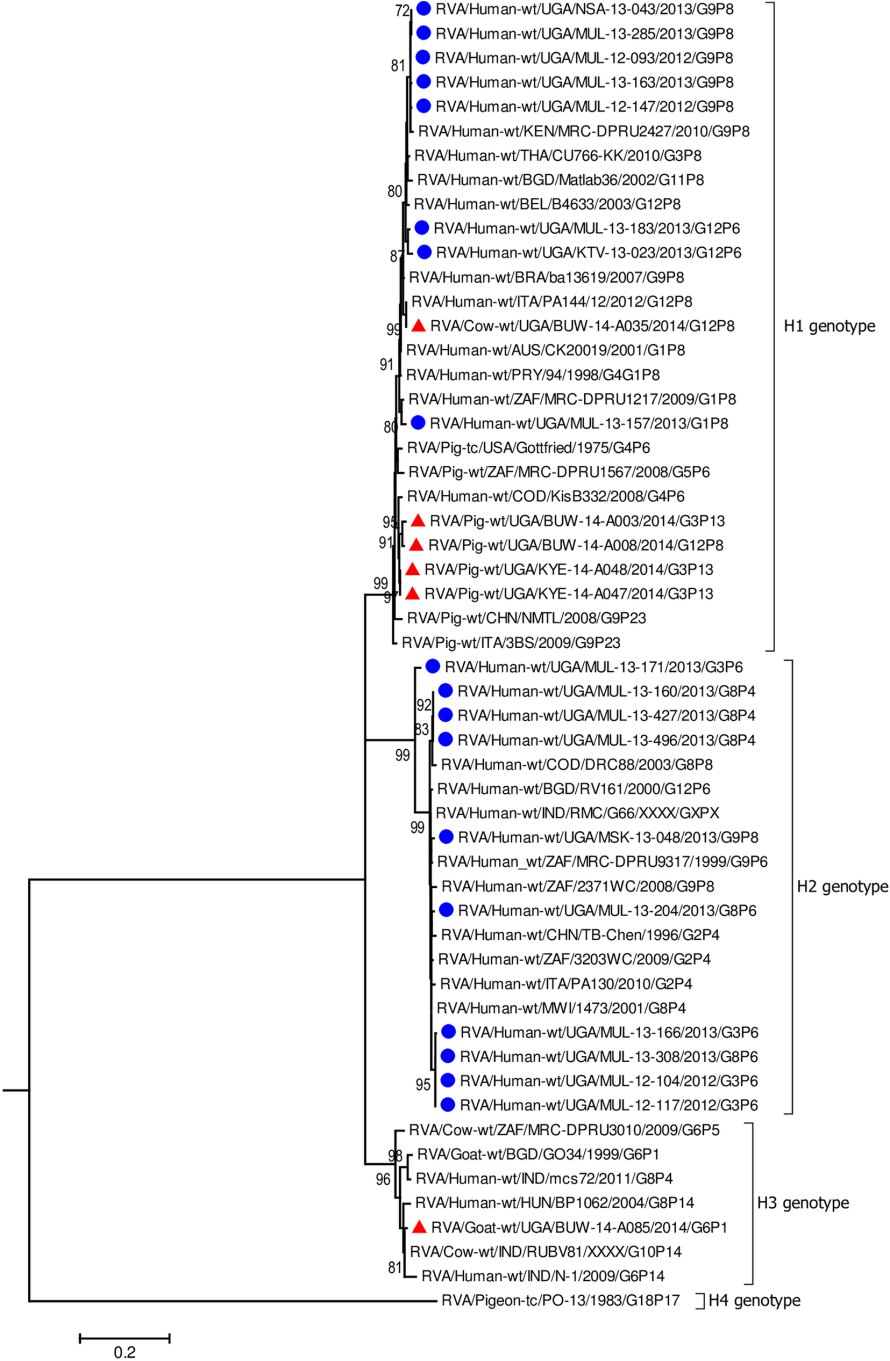
NSP5 gene (segment 11). Maximum Likelihood phylogenetic trees of nucleotide sequences of genome segment 11 of human and animal RVA strains circulating in Uganda, 2012–2014. Bootstrap values above 70 are shown for 1000 replicates. The Ugandan human strains are labelled with blue circles and the Ugandan animal strains with red triangles. Pigeon strain RVA/Pigeon-tc/PN/PO-13/1983/G18P[17] served as the outgroup. The scale bar at the bottom of the tree calibrates the genetic distance expressed as nucleotide substitution per site.

The nucleotide sequences of the genes derived from human RVAs were 82.2–100% identical to each other (S4 Table, S2 Table). Some genes of a few human strains were closely related to animal strains identified in this study or elsewhere. The VP1 gene sequences of MUL-13-204 clustered with the cogent genes of the goat strain GO34 with a nucleotide identity of 97% (Fig 2) [52]. The VP6 of human strain MUL-13-204 clustered with cogent genes of the porcine RVA strain HP140 which may be a bovine-human reassortant and caprine strain BUW-14-A085 and had 98% and 88.9% nucleotide identity, respectively (Fig 3) [53]. The nucleotide sequences of the NSP4 gene of MUL-13-204 clustered with the cogent gene of a caprine rotavirus strain GO34 and had 97% nucleotide identity (Fig 5) [52].

Some genes of the human strains were closely related to strains reported to have zoonotic origin. The VP2 gene sequences of MUL-13-204 and MUL-13-157 were closely related (99.2% and 91% nucleotide similarity, respectively), to that of human strain 1473 from Malawi, which is artiodactyl-like; a human-bovine reassortant strain (S1 Fig) [29].

The NSP4 gene sequences of MUL-13-183 and KTV-13-023 clustered with porcine-like human RVA strains KDH684 and KisB332 from Kenya and DRC, respectively, and had 99% and 98% nucleotide identity, respectively (Fig 5) [16, 50].

Among the animal strains, the bovine strain, BUW -14- A035 showed high identity with human RVA strains in all genes with nucleotide identities of 96.9% to 98.5% (Figs 2–8, S1–S4 Figs).

**Fig 7:**
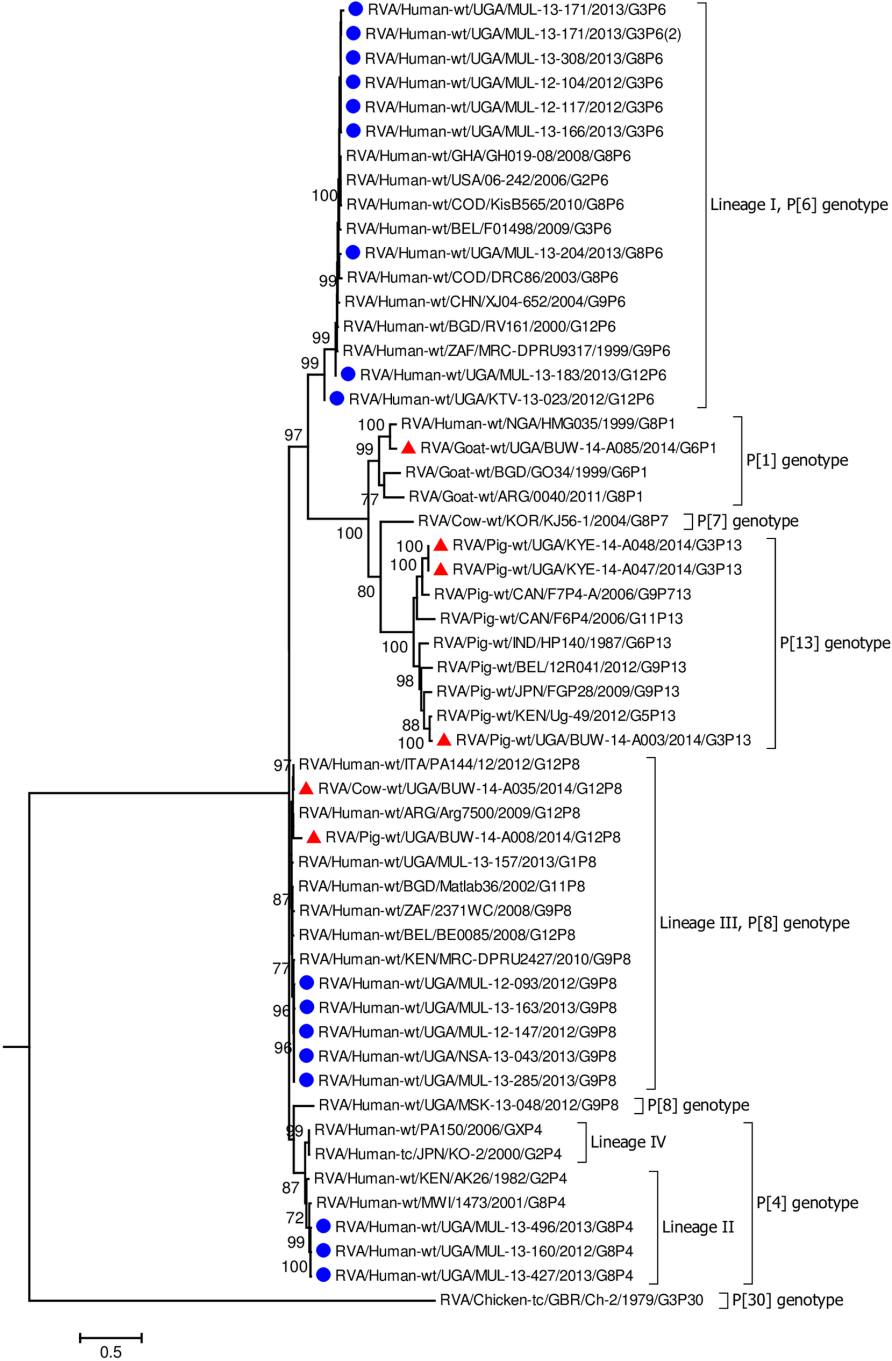
VP4 gene (segment 4). Maximum Likelihood phylogenetic trees of nucleotide sequences of rotavirus genome segment 4 of human and animal RVA strains circulating in Uganda, 2012–2014. Bootstrap values above 70 are shown for 1000 replicates. The Ugandan human strains are labelled with blue circles and the Ugandan animal strains with red triangles. Chicken strain, RVA/Chicken-tc/GBR/Ch-2/1979/G3P[30] served as the outgroup. The scale bar at the bottom of the tree calibrates the genetic distance expressed as nucleotide substitution per site.

**Fig 8:**
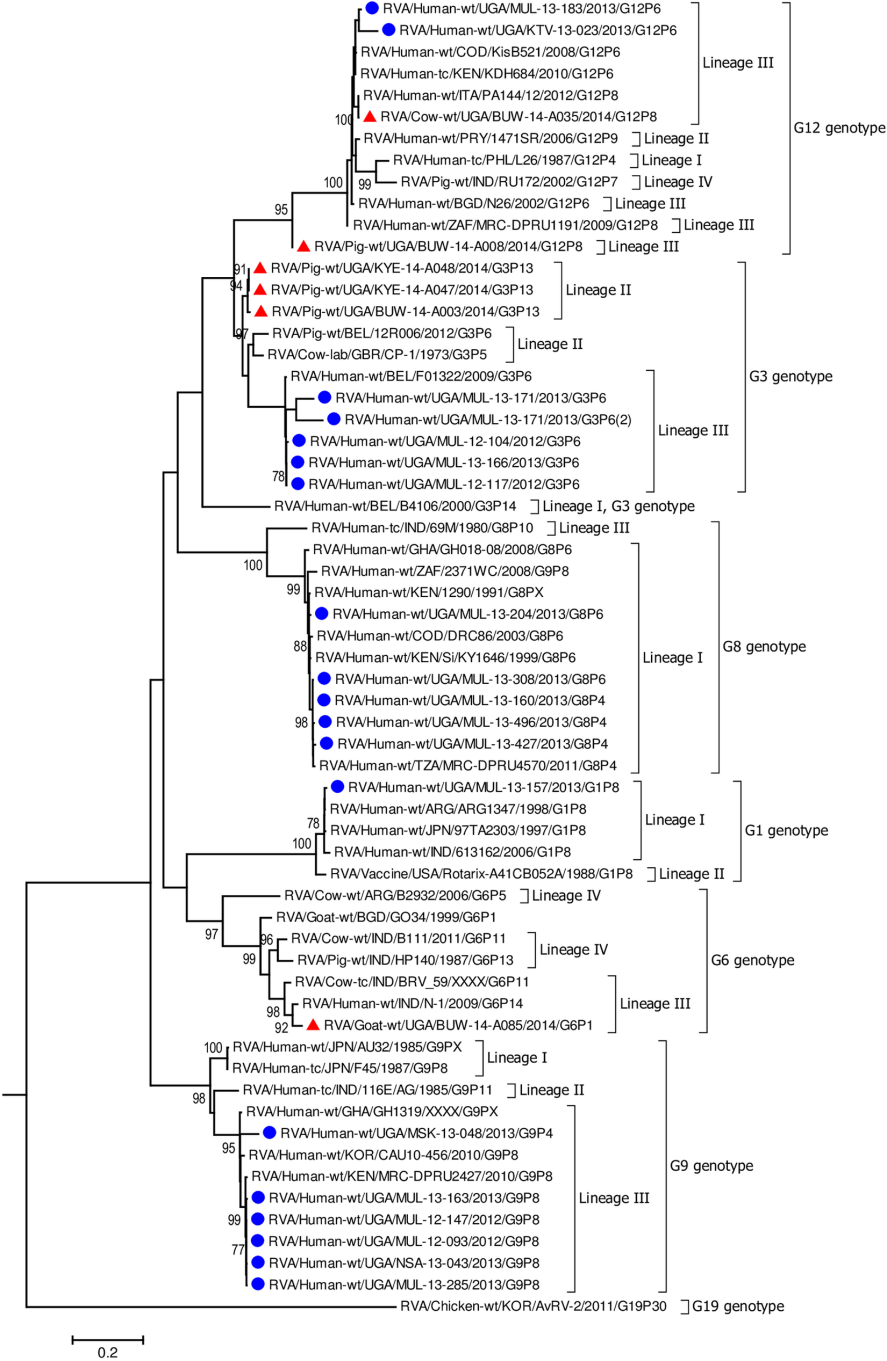
VP7 gene (segment 9). Maximum Likelihood phylogenetic trees of nucleotide sequences of rotavirus genome segment 9 of human and animal RVA strains circulating in Uganda, 2012–2014. Bootstrap values above 70 are shown for 1000 replicates. The Ugandan human strains are labelled with blue circles and the Ugandan animal strains with red triangles. Chicken strain RVA/Chicken-wt/KOR/ArRv-2/2011/G19P[30] served as the outgroup. The scale bar at the bottom of the tree calibrates the genetic distance expressed as nucleotide substitution per site.

Porcine strains KYE-14-A047 and KYE-14-A048 collected from the same homestead clustered together with nucleotide identity of 95.5–99.6% across all genes. Porcine strains BUW-14-A003 and BUW-14-A008 were also collected from the same household, and the nucleotide sequences of the genes: VP2, VP3, NSP2, NSP3, NSP4 and NSP5 clustered together (S1 Fig, S2 Fig, S4 Fig, Figs 4–6).

Each individual gene across the porcine strains clustered with porcine strains of this study and from elsewhere, and with strains that had been identified in humans but had evidence of zoonotic transmission (Figs 2–4, Fig 6, S1–S3 Figs) [16, 54–56]. The only exceptions were the genes encoding the VP7 and VP4 of strain BUW-14-008, which were more closely related to those from human strains (Fig 7, Fig 8) [57].

In addition, the NSP4 genes of the studied porcine strains clustered with the Ugandan human RVA strains: MUL-13-183 and KTV-13-023 (Fig 5).

None of the nucleotide sequences of the caprine strain BUW-14-A085 clustered with those of other animal and human sequences in this study, except the nucleotides sequences of the gene encoding VP6 which clustered with the human strain MUL-13-204 (Fig 3).

All the remaining genes of the caprine strain BUW-14-A085 clustered with those identified in other caprine strains or primarily shared among bovine and caprine strains. The NSP2 cluster, however, included cogent genes of RVAs from diverse animal species: caprine, bovine, pigs, dogs, sheep and antelope RVA strains (Fig 4). Sequences of VP1, VP2, VP3 and NSP5 genes of BUW-14 A085 clustered with human strains of zoonotic origin (Fig 2, Fig 6, S1 Fig, S2 Fig) [18, 53, 58, 59].

## Discussion

Interspecies transmission of rotaviruses is thought to occur frequently due to the close proximity or sharing of animal and human dwellings in some communities, particularly in low income countries [16]. In view of this, we sought to investigate whether interspecies transmission of RVAs was occurring and possibly contributing to the genetic diversity of RVA strains in Uganda.

The human rotaviruses analysed in the present study were closely related to RVAs from other parts of Africa including neighbouring countries: Kenya, Democratic Republic of the Congo (DRC) and Tanzania. Direct reassortment with porcine or porcine-like human RVAs such as those found in Kenya and DRC may potentially have led to the emerging of NSP4 genes in the human strains MUL-13-183 and KTV-13-023 [16, 50]. By contrast, the VP2 genes of MUL-13-204 and MUL-13-157 strains, because of their close relationship to human RVA strain 1473 from Malawi [29], may have a different origin. The VP6 genes of MUL-13-204 may have been a result of interspecies transmission and reassortment events. Although the NSP4 gene of the human strain MUL-13-204 was closely related to those of other human strains characterised in the present study, it clustered with the NSP4 gene of strain GO34, a caprine rotavirus strain from Bangladesh that is thought to be of bovine origin [52]. These findings highlight the challenges in identifying the geographical and temporal origin of such interspecies transmission through sequence analysis of data obtained in discrete cross-sectional studies.

In some of the RVA genes there was close similarity among the Ugandan human and animal strains. However, phylogenetic analysis of sporadic strains is not enough to elucidate where and when interspecies transmission and reassortment events took place. Nonetheless, in this study, the identification of one bovine strain (BUW-14-A035) in which all gene segments were highly related to human strains circulating in Uganda, was highly suggestive of a direct anthroponotic transmission event.

In order to establish the origin and, or timing of such RVA transmission events, future studies analysing large numbers of RVA strains collected from humans and other mammalian species over a longer period are warranted. The relative small number of samples is one limitation of this study. In addition, the human strains were all from moderate to severe cases of gastroenteritis. Therefore, zoonotic RVAs may have been missed if they circulate at low frequency or were associated with mild disease or asymptomatic infections. However, this study provides confirmation that RVAs causing moderate to severe diarrhoea in humans in Uganda are of common genotypes that have been detected globally and are primarily transmitted from person-to-person. Therefore rotavirus control measures targeting humans should be expected to significantly reduce RVA transmission and burden of moderate or severe rotavirus diarrhoea in Uganda.

Failure to detect two distinct variants for all 11 genes of human strain MUL-13-171 may be due to the variant strains sharing the same sequence in the remaining genes. Also the variants may represent drift and the accumulation of point mutations during the infection and shedding period. Although the presence of quasispecies is expected among RNA viruses, in the acute phase of infections minority species are present at very low levels [60]. It is potentially possible that we were only able to detect variants among those genes that through immune pressure may be driven to hyper variability, such as VP7 and VP4. The heterozygosity detected among the three genes may therefore be associated with prolonged shedding and selective pressure, or as discussed above, with mixed infection [35].

In this study, G12P[8] strains, commonly associated with infection in humans [13, 61, 62] were detected in a pig and a cow. Furthermore, the cow had recent history of diarrhoea. All 11 genes of the bovine RVA strain were of likely human origin due to their high degree of nucleotide identity to those of human RVA in this study and human RVAs strains collected elsewhere [41]. Whereas most interspecies transmission reports observed bovine to human transmission [58, 59], the present study found evidence of human to bovine RVA transmission in Uganda. Since the bovine RVA infection may have been associated with disease, this warrants further research on animal husbandry and feeding practices that may promote inter-species transmission of RVA in this region and drive the emergence of reassortant strains.

G12P[8] strains have been occasionally characterised in pigs, especially those living in close proximity with humans and cows [62, 63]. In this study, we identified one porcine G12P[8] RVA strain (BUW-14-A008) from a household that also housed cattle. Although cattle and human samples collected from this household were rotavirus negative at the time of sampling, it is conceivable that this strain, which had 5 genes (VP1, VP4, VP7, NSP1 and NSP4) closely related to those from human RVAs, may have been derived through human-to-animal transmission and reassortment, as G12P[8] RVAs have the potential to infect all three hosts in the household. In addition, a second porcine strain from the same household (BUW-14-A003: G3P[13]) showed that despite the differences in their VP4 and VP7 genotypes, some genes (VP2,VP3, NSP2, NSP3, NSP4 and NSP5) clustered closely suggesting possible complex exchanges of RVA genes between pigs and humans. A study on RVAs in pigs in East Africa showed possible human to pig transmission of RVAs, where P[8] strains were closely related genetically to human RVAs [64]. These finding are in agreement with the hypothesis on the common origin of porcine and Wa-like human RVAs by Matthijnssens J *et al* [18].

The high nucleotide identity between all gene segments of porcine strains KYE-14-A047 and KYE -14-A048 suggests that the two pigs were infected by the same strain and provides evidence of ongoing transmission within a household. Similar observations have been reported in South Africa, where five sequences of rotavirus isolates from two calves on the same farm had high nucleotide identity [65].

There are few reports on whole genome sequencing of caprine RVA strains [52, 66]. Our study found that a potentially complex series of reassortment events may have led to the origin of the caprine RVA strain BUW-14-A085 with RVA genes of potential human, bovine and porcine origin. This was similar to what was reported for a caprine RVA strain, GO34, in Bangladesh [52]. In Uganda, most of the animals live in close proximity with each other. This could explain the observed RVA reassortment events. The caprine strain, BUW-14-A085 had an overall genotype constellation of G6-P[1]-I2-R2-C2-M2-A11-N2-T6-E2-H3, which has been found in cattle [58, 67] and thus, may have originated through direct bovine-to-goat transmission.

## Conclusions

The present study shows occurrence of interspecies transmission of RVAs of human and animal origins in Uganda with possible reassortment among rotaviruses from different host species. Whereas previous reports on RVA evolution have been mainly on RVA transmission from animals to humans, this study suggests that domestic animals may also become infected by RVAs from humans. The complex reassortment events of rotaviruses from different host species may lead to the emergence of novel rotavirus strains with the potential to influence the epidemiology of rotaviruses in this setting. Therefore, continued surveillance of rotavirus strains from both animals and humans is necessary to monitor changes in the rotavirus epidemiology over time. Whole genome sequencing of rotaviruses from domestic animals and humans living in close proximity can increase our understanding of the molecular epidemiology and evolution of RVAs in Uganda and other countries. Such studies, if conducted in a systematic way, will help to elucidate the complex interspecies transmission patterns that lead to the diversity of rotavirus strains seen among the different species. Ultimately, this should lead to a better understanding of the genes or gene combinations that govern successful transmission between hosts or that are likely to result in host restriction.

## Supporting information

S1 Table Geographical Positioning System (GPS) coordinates of the hospitals at which the studied humans were recruited and households of the animals whose rotavirus samples were sequenced. (DOC)

S2 Table The NCBI GenBank accession numbers of the nucleotide and amino acid sequences generated in this study. (XLS)

S3 Table Reference sizes of genome segments of fully sequenced SA11 rotavirus strain, and the comparative sizes (percent sequence) of partially sequenced genome segments of the animal rotavirus strains of this study. (DOCX)

S4 Table Comparison of the characterised human and animal RVA samples in Uganda, 2012–2014: Accession numbers of species of the closest nucleotide sequence identity to the characterised humans. (XLSX)

S1 Fig VP2 gene. Maximum Likelihood phylogenetic trees of nucleotide sequences of rotavirus genome segment 2 encoding VP2 gene of human and animal strains circulating in Uganda, 2012–2014. Bootstrap values above 70 are shown for 1000 replicates. The Ugandan human strains are labelled with blue circles and the Ugandan animal strains are labelled with red triangles. Pigeon strain RVA/Pigeon-tc/PN/PO-13/1983/G18P[17] served as the outgroup. The scale bars at the bottom of the trees calibrate the genetic distance expressed as nucleotide substitution per site.
(TIF)

S2 Fig VP3 gene. Maximum Likelihood phylogenetic trees of nucleotide sequences of rotavirus genome segment 3 encoding VP3 gene of human and animal strains circulating in Uganda, 2012–2014. Bootstrap values above 70 are shown for 1000 replicates. The Ugandan human strains are labelled with blue circles and the Ugandan animal strains are labelled with red triangles. Pigeon strain RVA/Pigeon-tc/PN/PO-13/1983/G18P[17] served as the outgroup. The scale bars at the bottom of the trees calibrate the genetic distance expressed as nucleotide substitution per site.
(TIF)

S3 Fig NSP1 gene. Maximum Likelihood phylogenetic trees of nucleotide sequences of rotavirus genome segment 5 encoding NSP1 of human and animal strains circulating in Uganda, 2012–2014. Bootstrap values above 70 are shown for 1000 replicates. The Ugandan human strains are labelled with blue circles and the Ugandan animal strains are labelled with red triangles. Pigeon strain RVA/Pigeon-tc/PN/PO-13/1983/G18P[17] served as the outgroup. The scale bars at the bottom of the trees calibrate the genetic distance expressed as nucleotide substitution per site.
(TIF)

S4 Fig NSP3 gene. Maximum Likelihood phylogenetic trees of nucleotide sequences of rotavirus genome segment 7 encoding NSP3 of human and animal strains circulating in Uganda, 2012–2014. Bootstrap values above 70 are shown for 1000 replicates. The Ugandan human strains are labelled with blue circles and the Ugandan animal strains are labelled with red triangles. Pigeon strain RVA/Pigeon-tc/PN/PO-13/1983/G18P[17] served as the outgroup. The scale bars at the bottom of the trees calibrate the genetic distance expressed as nucleotide substitution per site.
(TIF)
