# Supplementary material for: Whole genome analysis of selected human and animal rotaviruses identified in Uganda from 2012 to 2014 reveals complex genome reassortment events between human, bovine, caprine and porcine strains
Source: PLoS One. 2017 Jun 22;12(6):e0178855. doi: 10.1371/journal.pone.0178855 (PMC5480867; doi:10.1371/journal.pone.0178855)
Supplement: S1 Table — (DOC) [file pone.0178855.s001.doc]

| **ID** | **Coordinates** |
| --- | --- |
| St Francis Hospital, Nsambya | N 00^0^18.018 |
|  | E 032^0^35.233 |
| Mulago National Referral Hospital | N 00^0^20.423 |
|  | E 032^0^34.527 |
| Kitovu Hospital | S 00^o^ 20.592 |
|  | E 031^0^45.574 |
| Masaka Regional Referral Hospital | S 00^0^19.795 |
|  | E 031^0^44.244 |
| BUW-14-A003  BUW-14-A008 | S 00^0^22.942 |
|  | E 031^0^46.130 |
| BUW-14-A035 | S 00^0^25.928 |
|  | E 031^0^50.351 |
| BUW-14-A085 | S 00^0^26.083 |
|  | E 031^0^46.998 |
| KYE-14-A047  KYE-14-A048 | S 00^0^35.319 |
|  | E 031^0^44.207 |

**S1 Table. Geographical Positioning System (GPS) coordinates of the hospitals at which the studied humans were recruited and households of the animals whose rotavirus samples were sequenced**.
