# Supplementary material for: Whole genome analysis of selected human and animal rotaviruses identified in Uganda from 2012 to 2014 reveals complex genome reassortment events between human, bovine, caprine and porcine strains
Source: PLoS One. 2017 Jun 22;12(6):e0178855. doi: 10.1371/journal.pone.0178855 (PMC5480867; doi:10.1371/journal.pone.0178855)
Supplement: S3 Table — (DOCX) [file pone.0178855.s003.docx]

| **S3 Table.** Reference sizes of genome segments of fully sequenced SA11 rotavirus strain, and the comparative sizes (percent sequence) of partially sequenced genome segments of the animal rotavirus strains of this study | | | | | | | | | | | |  |
| --- | --- | --- | --- | --- | --- | --- | --- | --- | --- | --- | --- | --- |
|  | | | | | |  |  |  |  | |  |  |
|  |  |  |  |  |  |  |  |  |  | |  |  |
| **Genome segments** | **VP1** | **VP2** | **VP3** | **VP4** | **VP6** | **VP7** | **NSP1** | **NSP2** | **NSP3** | | **NSP4** | **NSP5** |
| **Reference: genome segment size for fully sequenced Simian Rotavirus A SA11 strain (bp) ^c^** | 3302 | 2690 | 2591 | 2362 | 1356 | 1062 | 1611 | 1059 | 1105 | | 751 | 667 |
|  | **Genome segment size for partially sequenced animal rotavirus genomes (bp)** | | | | | | | | | | | |
| **Genome segment:** | **VP1** | **VP2** | **VP3** | **VP4** | **VP6** | **VP7** | **NSP1** | **NSP2** | **NSP3** | **NSP4** | | **NSP5** |
| **Study strain name** |  |  |  |  |  |  |  |  |  |  | |  |
| RVA/Goat-wt/UGA/BUW-14-A085/2014/G6P[1] | * | 2663  (98.9) | 2585  (99.8%) | 2300  (97.4%) | 1242  (91.6%) | 582  (54.8%) | 1586  (98.4%) | 1018  (96.1%) | 1072  (97.0% | 657  (87.5%) | | 610  (91.5%) |
| RVA/Pig-wt/UGA/BUW-14-A008/2014/G12P[8] | 2591  (78.5%) | * | 2537  (97.9) | 1719  (72.8%) | 1198  (88.3%) | 1025  (96.5%) | 533  (33.1%) | 1009  (95.3%) | 991  (89.7%) | * | | 623  (93.4%) |
| RVA/Pig-wt/UGA/BUW-14-A003/2014/G3P[13] | 2365  (71.6%) | 1169  (43.5%) | 2055 (79.3%) | 1686  (71.4%) | 1092  (80.5%) | 797  (75.0%) | 890  (55.2%) | 964  (91.0%) | 961  (87.0%) | 552  (73.5%) | | 602  (90.3%) |
| RVA/Pig-wt/UGA/KYE-14-A047/2014/G3P[13] | 3212  (97.3%) | * | 2576  (99.4%) | 2331  (98.7%) | * | 1002  (94.4%) | * | * | * | 734 (97.7%) | | 637  (95.5%) |
| RVA/Pig-wt/UGA/KYE-14-A048/2014/G3P[13] | 3224  (97.6%) | * | 2580  (99.5%) | 2358  (99.8%) | * | 1037  (97.6%) | 1587  (98.5%) | 1011  (95.5%) | 1070  (96.8%) | * | | 649  (97.3%) |
|  |  |  |  |  |  |  |  |  |  |  | |  |
| * Genome segment was fully sequenced thus size not shown  % represents percentage of expected gene length obtained for the incompletely sequenced genome segments | | | | | | | | | | | | |

**^c^**Estes M, Kapikian A. Rotaviruses. In: Knipe DM, Howley PM, Griffin DE, Lamb RA, Martin MA, Roizman B, Straus SE, editors. Fields Virology. 5th edition Kluwer Health/Lippincott, Williams and Wilkins; Philadelphia: 2007. pp. 1917–1974.
