# Supplementary figures and images for: Whole genome analysis of selected human and animal rotaviruses identified in Uganda from 2012 to 2014 reveals complex genome reassortment events between human, bovine, caprine and porcine strains
Source: PLoS One. 2017 Jun 22;12(6):e0178855. doi: 10.1371/journal.pone.0178855 (PMC5480867; doi:10.1371/journal.pone.0178855)

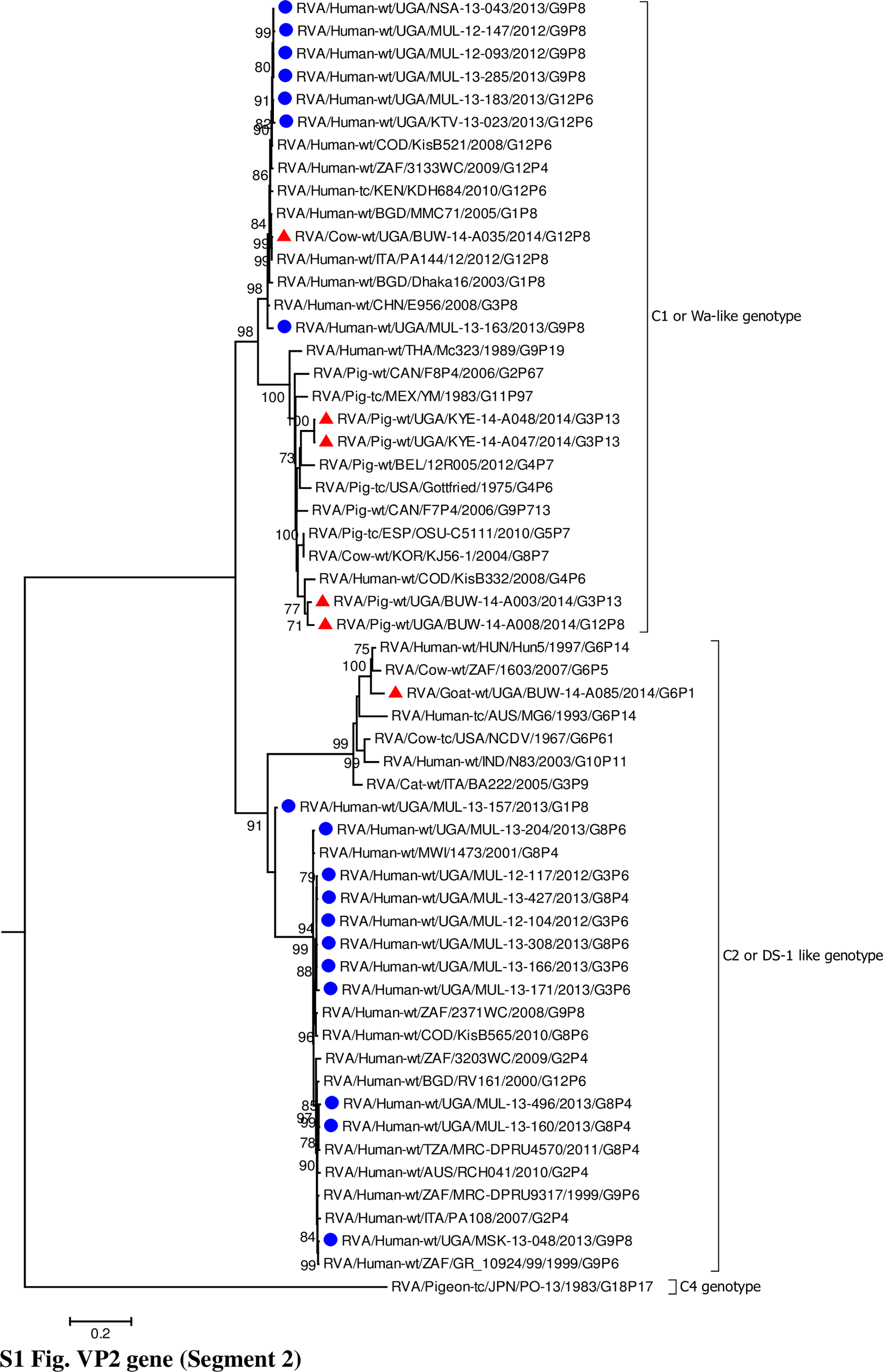

Supplement: S1 Fig — Maximum Likelihood phylogenetic trees of nucleotide sequences of rotavirus genome segment 2 encoding VP2 gene of human and animal strains circulating in Uganda, 2012–2014. Bootstrap values above 70 are shown for 1000 replicates. The Ugandan human strains are labelled with blue circles and the Ugandan animal strains are labelled with red triangles. Pigeon strain RVA/Pigeon-tc/PN/PO-13/1983/G18P[17] served as the outgroup. The scale bars at the bottom of the trees calibrate the genetic distance expressed as nucleotide substitution per site. (TIF) [file pone.0178855.s005.tif]

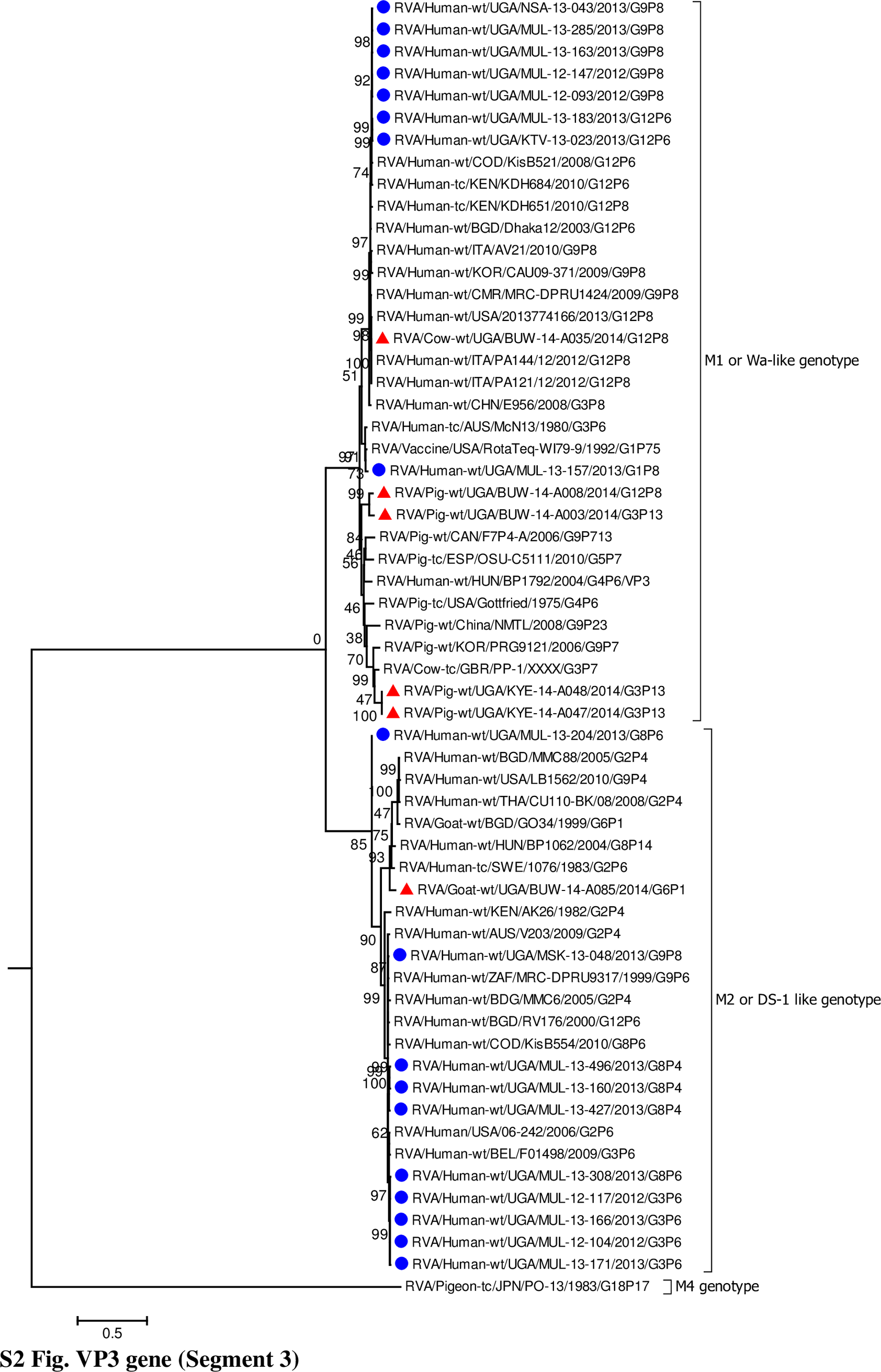

Supplement: S2 Fig — Maximum Likelihood phylogenetic trees of nucleotide sequences of rotavirus genome segment 3 encoding VP3 gene of human and animal strains circulating in Uganda, 2012–2014. Bootstrap values above 70 are shown for 1000 replicates. The Ugandan human strains are labelled with blue circles and the Ugandan animal strains are labelled with red triangles. Pigeon strain RVA/Pigeon-tc/PN/PO-13/1983/G18P[17] served as the outgroup. The scale bars at the bottom of the trees calibrate the genetic distance expressed as nucleotide substitution per site. (TIF) [file pone.0178855.s006.tif]

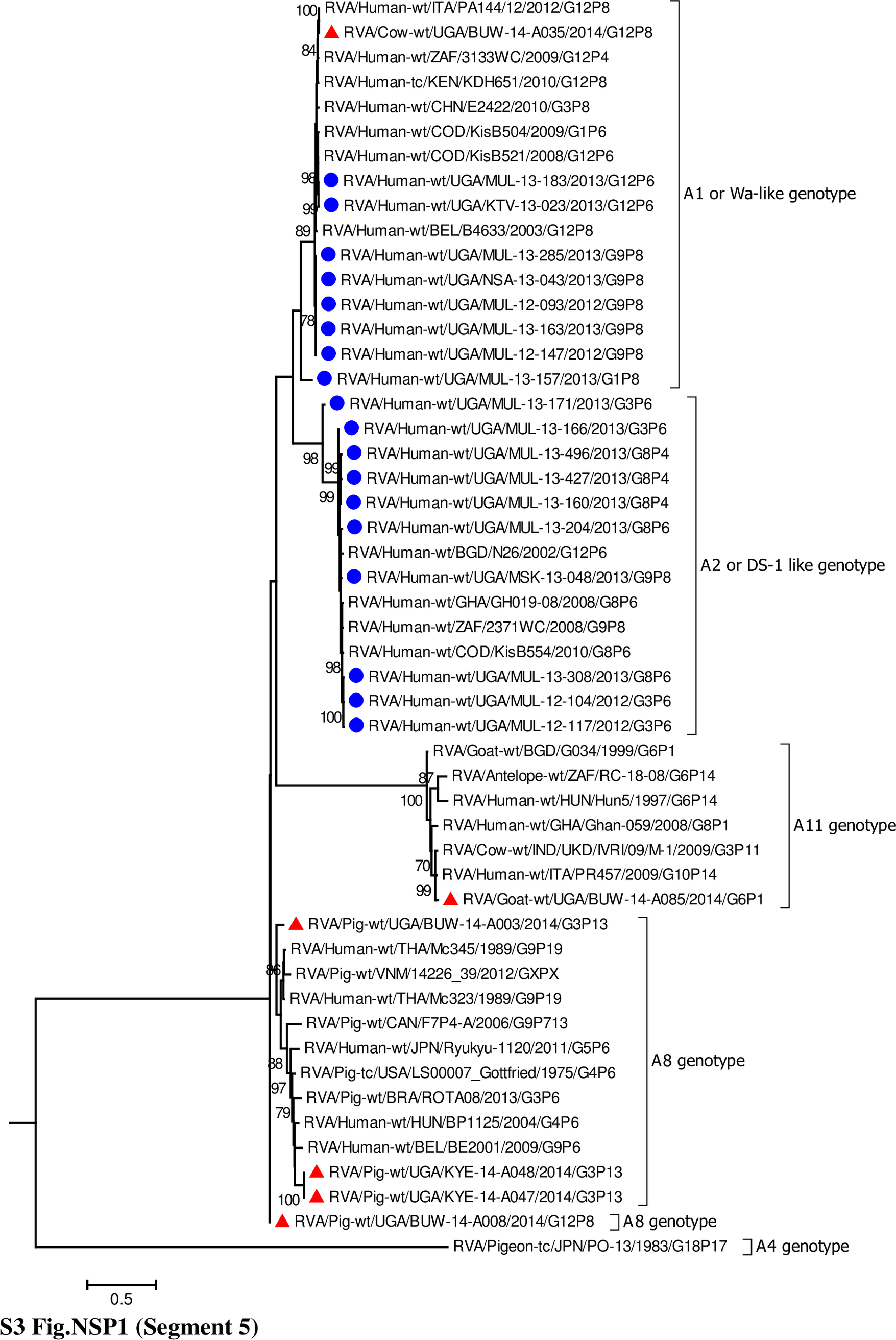

Supplement: S3 Fig — Maximum Likelihood phylogenetic trees of nucleotide sequences of rotavirus genome segment 5 encoding NSP1 of human and animal strains circulating in Uganda, 2012–2014. Bootstrap values above 70 are shown for 1000 replicates. The Ugandan human strains are labelled with blue circles and the Ugandan animal strains are labelled with red triangles. Pigeon strain RVA/Pigeon-tc/PN/PO-13/1983/G18P[17] served as the outgroup. The scale bars at the bottom of the trees calibrate the genetic distance expressed as nucleotide substitution per site. (TIF) [file pone.0178855.s007.tif]

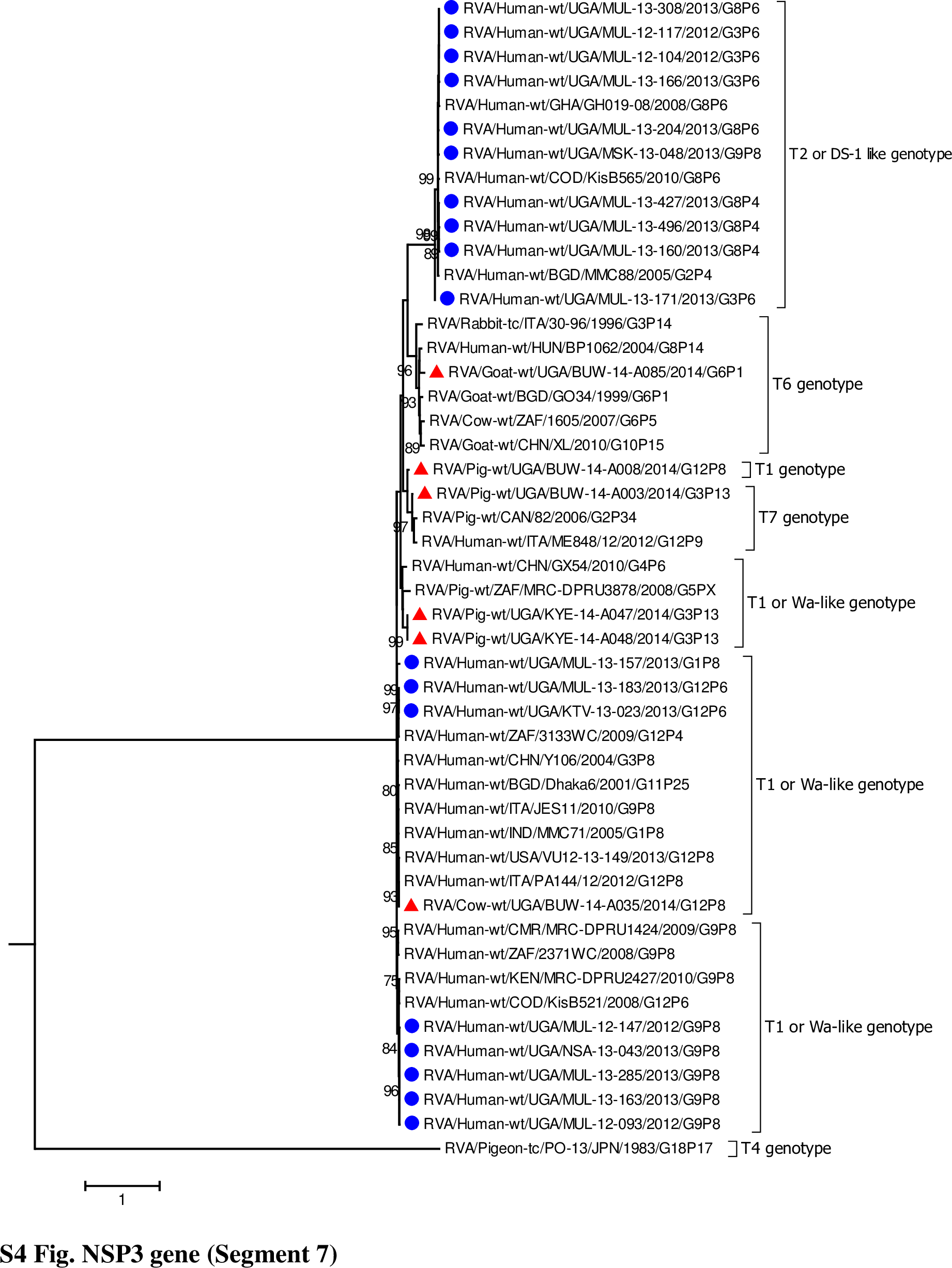

Supplement: S4 Fig — Maximum Likelihood phylogenetic trees of nucleotide sequences of rotavirus genome segment 7 encoding NSP3 of human and animal strains circulating in Uganda, 2012–2014. Bootstrap values above 70 are shown for 1000 replicates. The Ugandan human strains are labelled with blue circles and the Ugandan animal strains are labelled with red triangles. Pigeon strain RVA/Pigeon-tc/PN/PO-13/1983/G18P[17] served as the outgroup. The scale bars at the bottom of the trees calibrate the genetic distance expressed as nucleotide substitution per site. (TIF) [file pone.0178855.s008.tif]
